# Pro-angiogenic potential of human chorion-derived stem cells: *in vitro* and *in vivo* evaluation

**DOI:** 10.1111/jcmm.12051

**Published:** 2013-04-04

**Authors:** Mohd-Manzor N Fariha, Kien-Hui Chua, Geok-Chin Tan, Yun-Hsuen Lim, Abdul-Rahman Hayati

**Affiliations:** a Department of Pathology, Faculty of Medicine, Universiti Kebangsaan MalaysiaCheras, Kuala Lumpur, Malaysia; b Department of Physiology, Faculty of Medicine, Universiti Kebangsaan MalaysiaCheras, Kuala Lumpur, Malaysia; c Department of Obstetrics & Gynecology, Faculty of Medicine, Universiti Kebangsaan MalaysiaCheras, Kuala Lumpur, Malaysia; d Faculty of Medicine and Health Sciences, Universiti Sains Islam MalaysiaKuala Lumpur, Malaysia

**Keywords:** fetal stem cells, pro-angiogenic, quantitative PCR, vascular, ischemic disease

## Abstract

Human chorion-derived stem cells (hCDSC) were previously shown to demonstrate multipotent properties with promising angiogenic characteristics in monolayer-cell culture system. In our study, we investigated the angiogenic capability of hCDSC in 3-dimensional (3D) *in vitro* and *in vivo* angiogenic models for the purpose of future application in the treatment of ischaemic diseases. Human CDSC were evaluated for angiogenic and endogenic genes expressions by quantitative PCR. Growth factors secretions were quantified using ELISA. *In vitro* and *in vivo* vascular formations were evaluated by histological analysis and confocal microscopic imaging. PECAM-1^+^ and vWF^+^ vascular-like structures were observed in both *in vitro* and *in vivo* angiogenesis models. High secretions of VEGF and bFGF by hCDSC with increased expressions of angiogenic and endogenic genes suggested the possible angiogenic promoting mechanisms by hCDSC. The cooperation of hCDSC with HUVECS to generate vessel-like structures in our systems is an indication that there will be positive interactions of hCDSC with existing endothelial cells when injected into ischaemic tissues. Hence, hCDSC is suggested as the novel approach in the future treatment of ischaemic diseases.

## Introduction

Tissues require blood vessels for their supplies, particularly of nutrients and oxygen and at the same time there is the need to remove waste products 1. Arteries, veins and capillaries have similar basic features. From innermost layer to outwards the blood vessel is made of endothelial cells, basement membrane, pericytes and smooth muscles. However, they may differ in their gene expressions, histology or to a certain extent, their functions. Formation of new blood vessels (angiogenesis) results from a well-orchestrated process of endothelial cells proliferation and migration being regulated by factors that are produced by the surrounding cells and matrix 2.

In the event of severe ischaemia neovascularization will prevent irreversible damages 3. Vascular endothelial growth factor (VEGF) and basic fibroblast growth factor (bFGF) are angiogenic growth factors recently discovered which could be used by researchers in ‘therapeutic angiogenesis’ in severe ischaemic diseases 4–6. The safe use of growth factor genes or proteins in therapeutic angiogenesis is supported by pre-clinical data. Genes and cytokines are also being used in several clinical trials for the same purpose 7. Initial clinical trials on a small scale have been successful. However, larger randomized placebo-controlled trials did not show sufficient angiogenesis to support tissue function or alleviate the symptoms 7. Hence, the use of a single factor has its limitations. Following this, the treatment of ischaemic tissues has taken its direction towards cell-based therapeutic angiogenesis.

The role of various stem/progenitor cells in the mechanism of neovascularization needs to be ascertained. Each type of cell may differ in their roles in therapy. For example, bone marrow-derived stem cells (BMC) secrete pro-survival and pro-angiogenic paracrine factors which promote neovascularization to preserve the ischaemic myocardium 8, 9. The pro angiogenic factors produced by potential cells need to be identified and characterized through further studies. Some cells may contribute in a different way, by providing building blocks for the process of new vessels formation.

Hence, we investigated human chorion-derived stem cells (hCDSC) from placenta for its angiogenic properties through both *in vitro* and *in vivo* studies. Stemness properties of hCDSC have been discussed in our previous report 10. Our previous finding showed that hCDSC retained the multipotent potential even at later passage. It contains high clonogenic precursor with 1:30 CFU-F frequency at seeding density of 200 cells/cm^2^. The flow cytometric analyses showed that the hCDSC at various passages were positive for mesenchymal surface markers (CD90, CD9, CD44 and CD73) and MHC class I (HLA ABC) and were negative for haematopoietic markers (CD34 and CD117), leucocytes marker (CD45), endothelial marker (PECAM-1) and MHC class II (HLA DR DP DQ). Great multilineage potentials of stem cells from this source as well as the ability to sustain some of the crucial stem cells characteristics during expansion have encouraged us to further our research into the application of hCDSC. In this study, hCDSC were allowed to grow in a three dimensional construct and observations were made on the formation of vessel-like structures. Detail analysis on the ability of *in vitro* vessel formation was done by real time Polymerase Chain Reaction (real time PCR) based on the angiogenic and endogenic genes expression, quantification of angiogenic growth factors secreted and confocal live-imaging assessment of the interactions between hCDSC and human umbilical vein endothelial cells (HUVECS) when forming vessel-like structures.

## Materials and methods

### Human chorion-derived stem cells isolation and expansion

Isolation and expansion of hCDSC was performed as described in previous report 10. Briefly, small pieces of chorion were digested with 0.3% Collagenase type I (Gibco-Invitrogen, Grand Island, NY, USA) in a shaker incubator at 37°C for 1 hr. The digested tissue was centrifuged at 600 × *g*, for 10 min. to yield the cell pellet. The cells were resuspended in equal volumes of Ham's F12 and Dulbecco's Modified Eagle Medium (DMEM/F-12) supplemented with 10% foetal bovine serum (FBS), 1X Glutamax, 50 μg/ml Vitamin C and 1X Antibiotic-antimycotic (Gibco-Invitrogen) and cultured in T25 flasks (Falcon, BD Biosciences, San Jose, http://www.bdbiosciences.com). All cultures were maintained at 37°C in an incubator with 5% CO_2_.

### Three dimensional (3D) angiogenesis assay in fibrin-matrigel construct (FMC)

Whole blood was taken from a single donor and the plasma was separated through centrifugation (700 × *g*, 10 min.) and kept at −30°C until required for each experiment. Approximately 2000 cells/μl (P3 cells) of hCDSC was suspended in 150 μl of human plasma and 150 μl of growth factor reduced matrigel matrix (BD, USA) with the addition of 20 μl of 1 M CaCl_2_ (Sigma-Aldrich, St. Louis, MO, USA) and 30 μl of approtinin (Calbiochem, Darmstadt, Germany). The cells-plasma matrigel suspension was then poured into 1 cm diameter hole made of 2% agarose gel in a 6 well plate and allowed to solidify in the CO_2_ incubator for 15 min. After 15 min., 3 ml of normal medium was added into the 6 well plate containing the cells and fibrin-matrigel layer. In separate parallel samples, HUVECS alone and a mixture of hCDSC and HUVEC (2000 cells/μl) were also cultured in FMC for comparison.

### *In vitro* study

The FMC was maintained at 37°C in an incubator with 5% CO_2_ for 15 days. The culture medium was changed every 3 days. *In vitro* FMC of hCDSC, HUVECs and mix cells were subjected to histological analysis, quantification of VEGF and bFGF secretion and quantitative angiogenic and endogenic genes expressions. Histological analysis of *in vitro* construct of FMC (*n* = 6) was made using frozen sections. Briefly, FMC was placed onto a pre-labelled tissue base mould and covered with OCT (Optimal Cutting Temperature) compound (Gibco-Invitrogen). The FMC block was sectioned using the cryotome and the tissue sections were placed onto glass slides. These sections were kept in 75% ethanol solution prior to staining. Analysis for structure formation was carried out using standard Haematoxylin & Eosin (H&E) staining procedure and immunostaining for Platelet/endothelial cell adhesion molecule 1 (PECAM-1), von Willebrand factor (vWF) and Alpha smooth muscle actin (α-SMA).

### *In vivo* study

Empty FMC, Human CDSC-FMC, HUVECs-FMC and the mix cells-FMC were formed as mentioned above. The formed constructs were immediately implanted into the subcutaneous region of anaesthetized athymic mice (BioLASCO, Taiwan) by creating a pocket of 1 cm^2^ in size through skin incision. The handling and care of the animal was carried out according to the animal ethic's guidelines of Universiti Kebangsaan Malaysia. After 15 days of implantation, the hCDSC, HUVECs and the mix cells FMC were harvested for histological (H&E) and immunostaining (PECAM-1, vWF and α-SMA) analysis. Empty FMC was also stained with H&E as control.

### Immunostaining

**Fig. 1:**
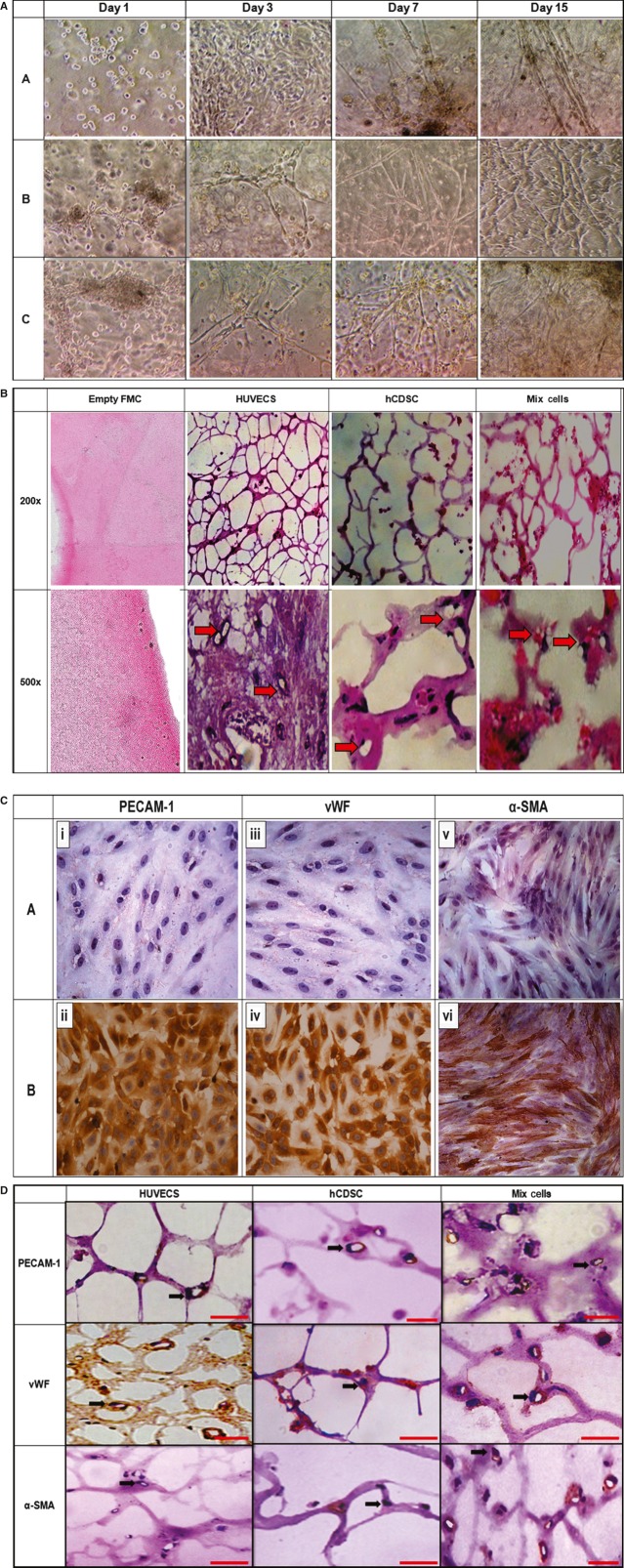
(**A**) Progressive development of blood vessel-like structures in three dimensional fibrin-matrigel construct (*in vitro*) with different groups of cells (A-HUVECS, B-hCDSC and C-mix culture of HUVECS-hCDSC) on day 1, 3, 7 and 15. Images were captured using Inverted microscope with 100× magnification. (**B**) Three dimensional fibrin-matrigel construct with different groups of cells harvested from *in vitro* experiment. The vascular-like networks formed by HUVECS, hCDSC and mix culture of HUVECS-hCDSC were stained by Haematoxylin & Eosin. (**C**) Reactions control for immuno-staining. A-Negative control (without primary antibodies, ×200) and B-positive control (with primary antibodies, ×200) for PECAM-1, vWF and α-SMA. HUVECS monolayer culture was used for PECAM-1 and vWF reactions control (i, ii, iii, iv) whereas hCDSC monolayer culture were used for α-SMA reactions control (v, vi). (**D**) Three dimensional fibrin-matrigel construct with different groups of cells harvested from *in vitro* experiment. Immunostaining of PECAM-1, vWF and α-SMA were performed on vascular-like network formed by HUVECS, hCDSC and mix cells (HUVECS and hCDSC) in the constructs *in vitro*. The arrow showed hematoxylin counterstained for the nucleus and the red scale bars represent the size of 30 μm.

Tissue sections placed on glass slides were treated with 3% hydrogen peroxide for 6 min. and incubated with 1% Bovine serum albumin (Sigma-Aldrich) solution at room temperature for 1 hr. Special treatment (1 hr incubation) using Rodent blocked M (Biocare Medical, Concord, CA, USA) was performed on *in vivo* tissue sections in order to block cross reaction of mice antigen. Diluted mouse anti-human PECAM-1 and α-SMA or rabbit anti-human vWF antibodies (DAKOCytomation) were applied to the slides for 1 hr. The slides were washed with TBS and incubated with antimouse or anti-rabbit secondary antibodies labelled polymer HRP (DAKOCytomation) for 30 min. at room temperature. The slides were washed and freshly prepared chromogen substrate (3,3′-diaminobenzidine) was applied for 7 min. Following another wash they were counterstained with Haematoxylin (Merck) for 2 min. Reactions control for PECAM-1 and vWF were performed on monolayer culture of HUVECs whereas reactions control for α-SMA was performed on monolayer culture of hCDSC (Fig. 1C).

### Quantification of VEGF and bFGF secretion in *in vitro* 3-D angiogenesis of fibrin-matrigel constructs (FMC)

The hCDSC-FMC, HUVECs-FMC and mix cells-FMC (*n* = 6 for each group) were prepared as described above. The culture media were replaced with fresh media on day 3, 6, 9, 12 and 15. The spent medium was collected for VEGF and bFGF quantification using Enzyme-linked immunosorbent assay (ELISA) according to the manufacturer's instruction (R&D System, Minneapolis, MN, USA).

### Total RNA extraction and quantitative polymerase chain reaction for angiogenic and endogenic genes

Fibrin-matrigel construct (*n* = 6 for each group) at day 3, 9 and 15 were harvested for total RNA extraction using TRI-Reagent (Molecular Research Center, Cincinnati, http://www.mrcgene.com) according to the manufacturer's instruction. Total RNA was stored at −80°C immediately after extraction. Complementary DNA was synthesized from 100 ng of Total RNA with SuperScript III reverse transcriptase (Invitrogen, Grand Island, NY, USA). The reaction was carried out according to the protocol recommended by manufacturer.

Quantitative PCR (qPCR) was performed using cDNA as template on the different groups of FMC to reveal the following angiogenic and endogenic genes expression levels namely the *VEGF, HGF, PGF, bFGF, Ang-1, vWF, VEGFR-2, ve-cadherin, PECAM-1, eNOS* and *CD34*. Detail procedures and the sequences for the primers used are as being previously described [Hayati *et al*., 2011; 11].

### *In vitro* confocal live imaging of 3-D angiogenesis assay

*In vitro* 3-D FMC of hCDSC, HUVECs and mix cells were formed as described above with the replacement of 6 well plate with 35 mm glass bottom dish (WillCo Wells B.V., Amsterdam, Netherlands). Prior to construct formation, cells were fluorescent-labelled using Qtracker® Cell Labeling Kit (Invitrogen) according to manufacturer's instructions. Qtracker® 525 (green colour) and Qtracker® 655 (red colour) were used to label hCDSC and HUVECs respectively. Image of hCDSC, HUVECs and mix cells FMC at day 3, 9 and 15 were captured using Nikon A1 Confocal microscope (Japan) and analysed using NIS-elements software (Nikon, Tokyo, Japan).

### Statistical analysis

Numeric data were expressed as mean ± standard error of mean (SEM). Differences in quantitative PCR (*n* = 6) and ELISA results (*n* = 6) between two groups were tested for significance using Student's *t*-test. A *P*-value <0.05 was considered to be significant.

## Results

### Histology of 3D construct-*in vitro*

In 3D angiogenesis assay; hCDSC and mixed cells groups were capable of forming vessel-like structures as in the HUVECS group (Fig. 1A). The development of vessels started with clustering of cells (day 1–day 2) followed by the formation of tubules which branched out to link the cell clusters (day 3–day 6). From day 7 to 11, more tubules were branching out from cell clusters to form capillary-like networks. Increasing complexities and lengths of the vessels were observed from day 12 to 15 with higher density of vessels being formed in the mixed cells constructs. Although hCDSC generated longer tubules, it was noticed that the tubular structures formed in the constructs tend to disrupt. Cross-sectional view demonstrated the formation of lumen in all types of constructs investigated (Fig. 1B). After 15 days of angiogenesis, immunostaining on the tissues harvested from all constructs were positive for PECAM-1 and vWF (Fig. 1D). However, only hCDSC and mixed cells constructs were positive for α-SMA.

### VEGF and bFGF secretions in 3D angiogenesis assay-*in vitro*

**Fig. 2:**
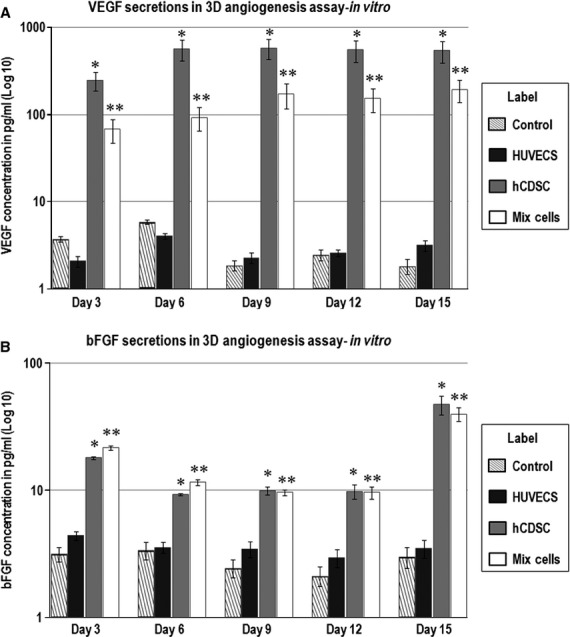
(**A**) VEGF secretion by HUVECS, hCDSC and mix cells (HUVECS and hCDSC) cultured in three dimensional fibrin-matrigel construct (*in vitro*) after 3, 6, 9 and 15 days of incubation. Culture medium for empty FMC culture was used as control. *Indicate significant difference when comparing hCDSC with control and HUVECS group while **indicate significant difference when comparing mix cells with control and HUVECS group (*P* < 0.05 and *n* = 6). (**B**) bFGF secretion by HUVECS, hCDSC and mix cells (HUVECS and hCDSC) cultured in three dimensional fibrin-matrigel construct (*in vitro*) after 3, 6, 9 and 15 days of incubation. Culture medium for empty FMC culture was used as control. *Indicate significant difference when comparing hCDSC with control and HUVECS group while **indicate significant difference when comparing mix cells with control and HUVECS group (*P* < 0.05 and *n* = 6).

Human CDSC demonstrated significantly (*P* < 0.05) higher VEGF (Fig. 2A) and bFGF (Fig. 2B) secretions when compared with the control and the HUVECS groups. Similar findings were observed when comparing mix cells with control and HUVECS group. However, the differences in both VEGF and bFGF secretions between hCDSC and mix cells (HUVECS + hCDSC) were not significant.

### Angiogenic and endogenic genes expression profile of hCDSC in FMC-*in vitro*

**Fig. 3:**
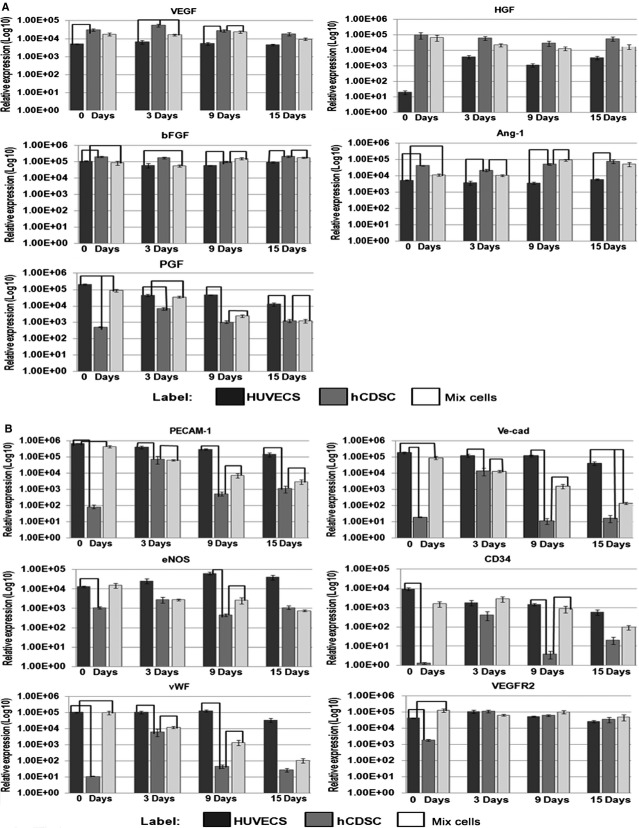
(**A**) Relative angiogenic genes expression level in HUVECS, hCDSC and the mix culture of HUVECS-hCDSC on day 0, 3, 9 and 15. The line indicate significant difference between two groups (*P* < 0.05 and *n* = 6). (**B**) Relative endogenic genes expression level in HUVECS, hCDSC and mix culture of HUVECS-hCDSC on day 0, 3, 9 and 15. The line indicate significant difference between two groups (*P* < 0.05 and *n* = 6).

The results demonstrated higher expressions of angiogenic genes; VEGF (6-fold), HGF 1275-fold, bFGF (2-fold) and Ang-1 (10-fold) by hCDSC FMC compared with HUVECS FMC. Angiogenic genes expressions were just slightly lower in mix cells FMC when comparing with hCDSC FMC. However, PGF were significantly (*P* < 0.05) higher in HUVECS and mix cells constructs than hCDSC FMC. There was no specific or consistent pattern of angiogenic genes expressions based on duration of culture (Fig. 3A).

From our findings, it was shown that hCDSC expressed lower endogenic genes especially at day 0. However, the expressions of PECAM-1, VE-cad, CD34 and vWF were markedly increased in hCDSC constructs harvested after 3 days of culture when compared to 0, 9 and 15 days whereas eNOS and VEGFR2 only slightly increased. The mix cells showed a decreasing pattern in the endogenic genes expressions throughout the culture period but from statistical analysis no significant differences were recorded when comparing groups at different point of time (Fig. 3B).

### Interactions of hCDSC and HUVECS in 3D angiogenesis assay-*in vitro*

**Fig. 4:**
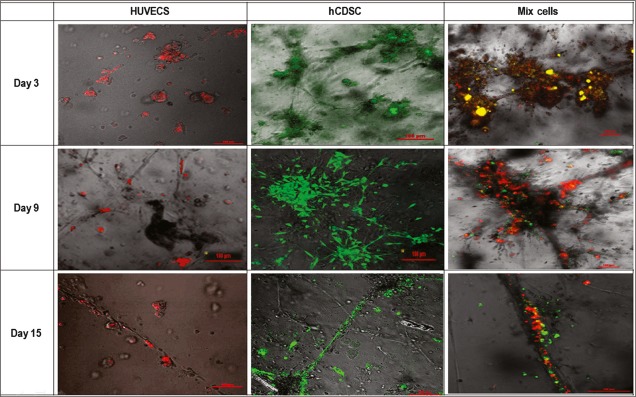
Live imaging of hCDSC (green) and HUVECS (red) co-culture in fibrin-matrigel construct after 3, 9 and 15 days. The overlapping of hCDSC and HUVECS were marked by yellow colour.

When we mixed hCDSC with HUVECS in 3D constructs and tracked the cells through live imaging confocal microscopy, it was shown that both types of cells were present in the vascular-like structures (Fig. 4). Cell clusters comprising of both hCDSC and HUVECS were noticeable on day 3. On day 9 and 15, the vascular network increased in density and length, the size of the vessels formed increased by 2 μm and the cooperation of both types of cells were more obvious.

### *In vivo* evaluation

**Fig. 5:**
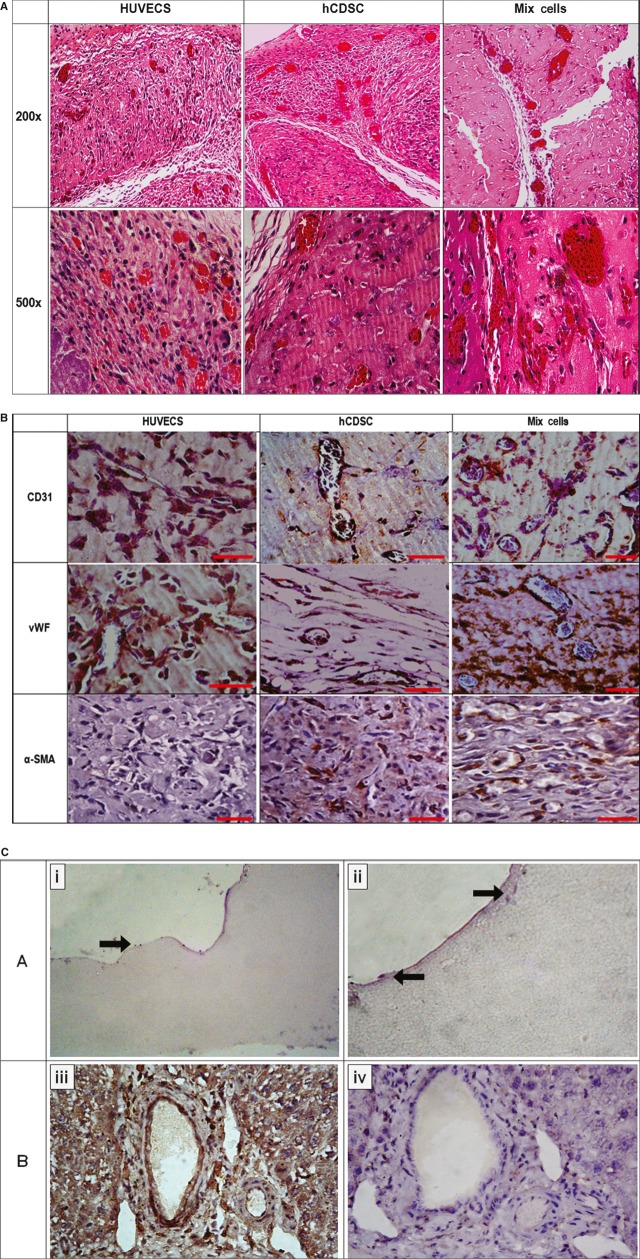
(**A**) Three dimensional fibrin-matrigel construct with different groups of cells harvested from *in vivo* experiment. The vascular-like networks formed by HUVECS, hCDSC and mix culture of HUVECS-hCDSC were stained by Haematoxylin & Eosin. (**B**) Three dimensional fibrin-matrigel construct with different groups of cells harvested from *in vivo* experiment. Immunostaining of PECAM-1, vWF and α-SMA were performed on vascular-like network formed by HUVECS, hCDSC and mix cells (HUVECS and hCDSC) in the three dimensional fibrin-matrigel construct *in vivo*. The red scale bars represent the size of 30 μm. (**C**) H&E staining of empty FMC harvested from *in vivo* experiment (A-i, ×100 and A-ii, ×200). The arrow showed infiltration of mouse cells. Mouse vessels stained with PECAM-1 antibody, with (B-iv, ×100) and without (B-iii, ×100) Rodent block M blocking reaction.

As shown by HUVECS, hCDSC constructs were also capable of forming vascular structures *in vivo*. The presence of red blood cells in the majority of the vessels indicates that the vessels are functioning (Fig. 5A). There was a relative increase each in the number and the size of vessels in the hCDSC and HUVECS co-culture as comparedwith hCDSC alone. Most of the vessels showed an endothelial lining with very thin or absence of the smooth muscle layer, the latter being structures of capillaries or small venules. Immunohistochemistry showed positive expressions of PECAM-1, vWF and α-SMA in all types of constructs except for HUVECS which were positive for PECAM-1 and vWF only (Fig. 5B). Figure 5C-panel B showed that the Rodent Block M reactions had successfully prevented cross reaction of the antibody being used with the host's cells. Infiltration of the host's cells into the periphery of the empty FMC was observed on H&E staining (Fig. 5C-A).

## Discussions

Through our investigations, the formation of vessels in hCDSC 3D constructs in both *in vitro* and *in vivo* is partly influenced by the secretion of known potent angiogenic growth factors; VEGF and bFGF. Differentiation of hCDSC into endothelial-like cells (PECAM-1^+^ cells) is likely the result of stimulation by VEGF. The proliferation and migration of these new endothelial cells to form vascular network might have been further induced by both VEGF and bFGF. It was reported that VEGF and bFGF have synergistic effects resulting in improved collateral circulation with significant hemodynamics in both *in vitro* and *in vivo* system 12, 13. The high secretion of both growth factors by hCDSC will be important in regulating angiogenesis for the treatment of ischaemic tissue as it was shown that administration of a single angiogenic growth factor in patients with atherosclerotic disease had insufficient effect 14, 15. Based on the pattern of secretion of growth factors in Figure 2, we hypothesized that part of the population of hCDSC had differentiated to endothelial cells in the absence of HUVECS,, whereas others remained as mesenchymal phenotype secreting high levels of VEGF and bFGF. However, in the co-cultured cells, the VEGF and bFGF that were secreted bound to receptors on endothelial cells resulting in decreased detection of both molecules in the medium.

Although quantification of other angiogenic molecules was not performed, the expressions of some angiogenic genes could address the additional angiogenic properties of hCDSC. Higher expressions of HGF and Ang-1 in hCDSC compared with HUVECS suggested the potential effect of hCDSC secretions in activating endothelial cells and promoting vascular maturation 16. Meanwhile, the expressions of endogenic genes; PECAM-1, ve-cad, eNOS, CD34, vWF and VEGFR-2 which increased after 3 days culture in fibrin-matrigel matrix indicated possible changes towards endothelial cells phenotype. Except for VEGFR-2 expressions, other endogenic genes showed inexplicable decreasing pattern after day 3. The maintenance of VEGFR-2 might be due to the stimulation by bFGF in order for VEGFR-2 to respond to VEGF 17.

The mixture of fibrin and matrigel matrix (growth factor reduced) for the construct formation provides a scaffold for angiogenesis and is stimulatory for cell migration. The invasion and migration of cells require degradation of the extracellular matrix by proteolytic enzymes. The sprouting of vascular-like network in hCDSC constructs as well as tubular lumen formation indicate the possibility that hCDSC is capable of producing adequate matrix proteolytic enzymes.

Mural cells stabilize the process of blood vessel maturation. Bone marrow, peripheral blood and vessel wall—derived cells which are autologous and cord blood from allogeneic sources as vascular progenitor cells have been identified and tested in preclinical models for this purpose 18. It has previously been shown that the co-culture of adipose stromal cells and endothelial cells demonstrated the formation of denser and more stable vascular network 19 and this is consistent with the observations in our study using hCDSC and HUVECS. In comparison to mix cells and HUVECS, construct with hCDSC alone generated longer tubule *in vitro*. However, they lacked in stability and strength whereby the structure tend to disrupt. This might be due to the properties of adult stem cells which have preferences towards being supporting cells or smooth muscle cells rather than differentiating into mature endothelial cells. Although the possibility of hCDSC to undergo endothelial differentiation is proven (Fig. 1B), the important roles of hCDSC to stabilize and promote vessel maturation are more eminent 20–22. Therefore, by using dual-cell system, the generation of adequate neovascularization could make it more sufficient than just using the endothelial cells as the single cell source. However, the number of cells needed to generate optimum vessel sprouting should be further optimized in future experiment as the importance of this factor has been proven in previous report 19.

Dual-properties of hCDSC were observed in *in vivo* construct. The cells were able to differentiate into endothelial cells (PECAM-1^+^ vessels) as well as perivascular cells (α-SMA+ vessels) when cultured alone or combined with HUVECS. The presence of blood cells in the vessels suggests that the transplanted hCDSC had at least facilitated the formation of a functional blood vessel for circulation in the implant. MSC from various organs including placenta have been identified to have originated from perivascular cells/pericytes 23. This finding could explain the same properties shown by hCDSC when implanted subcutaneously in the nude mice. Unlike adipose-derived stem cells which only have phenotypic and functional properties equivalent to pericytes and promote angiogenesis through this manner 24, hCDSC might be involved in angiogenesis by promoting, generating as well as stabilizing the formed vessels. Although the vascular structures in the *in vivo* hCDSC-FMC and mix cells-FMC showed less or no smooth muscle layer formation; the constructs were positive for α-SMA immunostaining. This observation might be due to the assumption that the hCDSC had just differentiated into smooth muscle like cells but had not formed the smooth muscle layer of the vessel as yet within the 15 days FMC, thus the cells are positive for α-SMA but less/no smooth muscle layer can be seen in the tissue. However, further investigation is needed especially in understanding the role of hCDSC in ischaemic disease models.

Various angiogenic molecules 25, 26, endothelial cells and bone marrow stromal cells 27, 28 as well as other adult stem cells as adipocytes have been used in researches in the field of therapeutic angiogenesis 29. We had revealed the potential of hCDSC from human placenta for future pro-angiogenic therapy. These abundantly available and less controversial multipotent stem cells can be easily cultured and expanded to achieve the numbers required for treatment 10. Therefore the remarkable angiogenic characteristics shown by hCDSC can be applied in autologous cells transplantation for the treatment of ischaemic problems with optimal efficacy and at a minimal cost. Isolation and characterization of foetal stem cells for various applications are progressively emerging 30, 31. When focusing on endogenic and angiogenic characterization, besides hCDSC, endogenic cells derived from human cord blood and Wharton's jelly have also demonstrated the potentials towards vascular regeneration 32, 33.

Promising angiogenic and endogenic properties have been shown by hCDSC in both *in vitro* and *in vivo* models. Formation of vascular structures with positive markers for PECAM-1, vWF and α-SMA proved the capability of hCDSC to undergo angiogenesis. Furthermore, high secretions of potent angiogenic growth factors, VEGF and bFGF as well as high expressions of angiogenic genes in hCDSC support the great potential of these cells in pro-angiogenic therapy.
